# Potentials and Limitations of Real-Time Elastography for Prostate Cancer Detection: A Whole-Mount Step Section Analysis

**DOI:** 10.1100/2012/193213

**Published:** 2012-12-31

**Authors:** Daniel Junker, Georg Schäfer, Friedrich Aigner, Peter Schullian, Leo Pallwein-Prettner, Jasmin Bektic, Wolfgang Horninger, Ethan J. Halpern, Ferdinand Frauscher

**Affiliations:** ^1^Department of Radiology, Medical University of Innsbruck, Anichstraß 35, 6020 Innsbruck, Austria; ^2^Department of Pathology, Medical University of Innsbruck, Anichstraß 35, 6020 Innsbruck, Austria; ^3^Department of Urology, Medical University of Innsbruck, Anichstraß 35, 6020 Innsbruck, Austria; ^4^Department of Radiology, Hospital of the Sisters of Charity, 4020 Linz, Austria; ^5^Thomas Jefferson Prostate Diagnostic Center, Thomas Jefferson University, Philadelphia, PA 19107, USA

## Abstract

*Objectives*. To evaluate prostate cancer (PCa) detection rates of real-time elastography (RTE) in dependence of tumor size, tumor volume, localization and histological type. *Materials and Methods*. Thirdy-nine patients with biopsy proven PCa underwent RTE before radical prostatectomy (RPE) to assess prostate tissue elasticity, and hard lesions were considered suspicious for PCa. After RPE, the prostates were prepared as whole-mount step sections and were compared with imaging findings for analyzing PCa detection rates. *Results*. RTE detected 6/62 cancer lesions with a maximum diameter of 0–5 mm (9.7%), 10/37 with a maximum diameter of 6–10 mm (27%), 24/34 with a maximum diameter of 11–20 20 mm (70.6%), 14/14 with a maximum diameter of >20 mm (100%) and 40/48 with a volume ≥0.2 cm^3^ (83.3%). Regarding cancer lesions with a volume ≥ 0.2 cm³ there was a significant difference in PCa detection rates between Gleason scores with predominant Gleason pattern 3 compared to those with predominant Gleason pattern 4 or 5 (75% versus 100%; *P* = 0.028). *Conclusions*. RTE is able to detect PCa of significant tumor volume and of predominant Gleason pattern 4 or 5 with high confidence, but is of limited value in the detection of small cancer lesions.

## 1. Introduction

Diagnosis and therapy of prostate cancer (PCa) are discussed controversially. On the one hand, prostate-specific antigen (PSA) testing has low specificity for PCa detection and systematic biopsy low sensitivity, and on the other hand detection of insignificant PCa may lead to overdiagnosis and overtherapy with its cost and complications [1–3]. Strategies like active surveillance, watchful waiting, and focal therapy of index cancer lesions are becoming more popular [3, 4].

Imaging modalities like magnetic resonance imaging (MRI), novel transrectal ultrasound (TRUS) technologies, that is, contrast enhanced TRUS (CE-TRUS), or real-time elastography (RTE) and computer aided analysis of TRUS signals (i.e., HistoScanning or computerized TRUS with artificial neural network analysis) have shown to be helpfully in urological management of diagnosis and/or therapy strategies for PCa [5–8]. One of the key requirements of imaging is to demonstrate significant cancer lesions in the prostate with high confidence, since they may determine the clinical prognosis [4, 9]. Targeted biopsy, focal therapy, and therapy monitoring of these lesions then could become possible. Based on the tumor volume significant lesions are defined to be ≥0.2 cm^3^ or ≥0.5 cm^3^ [10, 11].

One possibility for visualization of PCa is the representation of tissue elasticity. Usually cancers have a higher cell and vessel density than normal tissue and are therefore associated with a decreased elasticity [12, 13]. This is similar to the digital rectal examination (DRE) of the prostate performed by the urologists, where hard palpable areas are classified as suspicious for PCa. However, only the posterior parts of the prostate can be evaluated by DRE [14]. RTE, an ultrasonic method which is able to demonstrate tissue elasticity color-coded, does not have this problem, since all anatomical regions of the peripheral zone (PZ) can be evaluated [6]. Furthermore, this noninvasive technique is time-and cost-effective and proved its potentials in PCa detection with promising results in former studies [14, 15]. In contrast to static MRI, targeted biopsy and focal therapy of the prostate can be done under real-time conditions with RTE. 

The aim of this study was to evaluate PCa detection rates of RTE in dependence of tumor size, tumor volume, localization, and histological type and to determine reasons for false negative findings.

## 2. Materials and Methods

### 2.1. Patients

From April 2010 to November 2011 39 consecutive patients with a median age of 63 years (range: 48–75 years) and a median serum PSA value of 5 ng/mL (range: 2.1–14 ng/mL) participated in this prospective single-center study. All patients were informed about the study design and the study objective. Written informed consent and a positive vote by the local ethics committee of Innsbruck were present. Men with biopsy proven PCa and who were scheduled for radical prostatectomy (RPE) were included. All participants underwent RTE before RPE, and after RPE the prostates were prepared as whole-mount step sections, and the boarder of cancer lesions were marked. DRE was not part of the study.

### 2.2. Real-Time Elastography

**Figure 1:**
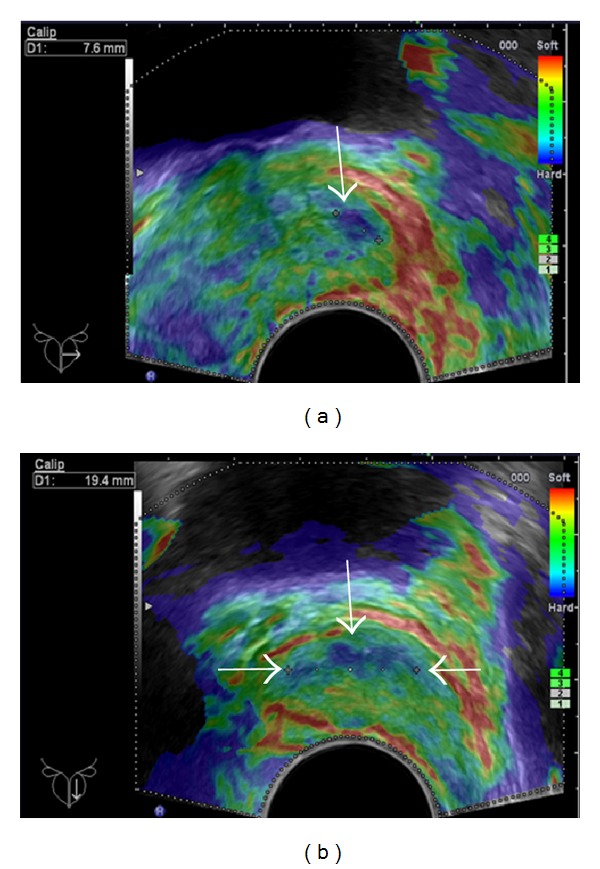
Hard area PZ mid-gland left measured with 7.6 mm in the axial plane ((a), white arrow) and with 19.4 mm in the sagittal plane ((b), white arrows).

RTE was done by one experienced uroradiologist (F. Aigner) on a EUB 8500 Hitachi ultrasound unit (Hitachi medical systems, Tokyo, Japan) using a 7.5 MHz end fire transrectal probe to assess tissue elasticity. Elastograms were obtained by slight prostate compression and decompression, which was manually induced by the investigator using the transrectal probe and controlled by the compression indicator on the monitor. Hard areas were considered PCa suspicious and color coded blue (Figure 1). These areas were reproducible in the axial and sagittal planes using a previously described approach [16]. Imaging findings suspicious for PCa were assigned to anterior, posterior, right, and left parts of the peripheral zone (PZ) of the prostate only, since most cancers originate from this zone and furthermore, transitional zone cancers are more likely to be less aggressive [17, 18].

### 2.3. Histopathology: Preparation, Reporting, and Correlation with RTE Findings

After RPE and fixation, the prostatectomy specimens were laminated in 4 mm thick slices with an orientation of 90° to the urethra and prepared according to the Stanford protocol. Pathological analysis was performed by one dedicated uropathologist (G. Schäfer), who outlined every cancer lesion and reported an assigned Gleason score. Tumor measures were provided in consideration of a shrinkage factor. The whole-mount step sections have been scanned in our system and were used in digital form for a correlation with the data of imaging findings. The PZ was divided in anterior, posterior, right, and left parts and the limit between anterior and posterior part was defined as the section running through the widest transverse diameter of the prostate. 

Based on histopathology, the lesions were classified according to their maximal diameter in the following 4 categories: lesions with a maximum diameter of 0–5 mm, 6–10 mm, 11–20 mm, and >20 mm. Furthermore, lesions were classified to their tumor volume in the following 2 categories: lesions with a volume of ≥0.2 cm^3^ and ≥0.5 cm^3^.

### 2.4. Statistical Analysis

Cancer detection rates based on tumor size and tumor volume as well as patient characteristics were summarized with frequencies and percentages or with median, range, minimum, and maximum values. The chi-square test was used to calculate significant differences between PCa detection rates based on localization, prostate volume (PV), tumor size, Gleason Scores, and serum PSA values. All statistical calculations were performed using SPSS 18.0 software, and a *P* < 0.05 was considered statistically significant.

## 3. Results

Overall, histological examination of the 39 prostatectomy specimens showed 147 cancer lesions in the PZ with a median size of 7.7 mm (range: 2–30.8 mm) of which 43 (29.3%) were localized in the anterior part, 90 (61.2%) in the posterior part, and 14 (9.5%) in both anterior and posterior parts of the prostate. RTE detected a total of 54 cancer lesions out of the 147 (36.7%). The median volume when only including tumors ≥0.2 cm^3^ was 0.85 cm^3^ (range: 0.21–11.18 cm^3^). The median Gleason score of all cancer lesions was 6 (range: 5–10) and of cancer lesions ≥0.2 cm^3^ was 7 (range: 6–10).

### 3.1. PCa Detection Rates in Dependence of Tumor Size (Figure 2)

RTE detected 6 of 62 cancer lesions with a maximum diameter of 0–5 mm (9.7%), 10 of 37 with a maximum diameter of 6–10 mm (27%), 24 of 34 with a maximum diameter of 11–20 mm (70.6%) and, 14 of 14 with a maximum diameter of >20 mm (100%).

### 3.2. PCa Detection Rates in Dependence of Tumor Volume (Figure 3)

RTE detected 40 of 48 cancer lesions with a tumor volume ≥0.2 cm^3^ (83.3%) and 31 of 34 with a tumor volume ≥0.5 cm^3^ (91.2%).

### 3.3. PCa Detection Rates Including all Cancer Lesions ≥0.2 cm^3^ in Dependence of Localization, Gleason Scores, Prostate Volumes, and PSA Values (Table 1)

RTE detected 6 of 9 cancer lesions in the anterior part (66.7%; group 1), 20 of 25 cancer lesions in the posterior part (80%; group 2), and 14 of 14, if the cancer lesions were located in both anterior and posterior parts (100%; group 3). PCa detection was not significantly different between group 1 and 2 (*P* = 0.419).

There was no significant difference for PCa detection in prostate volumes <40 mL and ≥40 mL (*P* = 0.204) and at serum PSA values <4 ng/mL and ≥4 ng/mL (*P* = 0.885).

A significant difference in PCa detection was found for PCa with a predominant Gleason pattern ≤3 and ≥4 (*P* = 0.028).

### 3.4. False Negative Findings on RTE for Cancer Lesions ≥0.2 cm^3^

All 8 missed cancer lesions ≥0.2 cm^3^ had a predominant Gleason pattern of 3. Six of these eight lesions had sparse architecture on histology, which is despite the malignant components composed of normal glands and glands with dilated lumina. Only two lesions were dense and had tumor volumes of 0.41 cm^3^ and 0.22 cm^3^, respectively.

## 4. Discussion

A total of 26% (10/39) of our study population had serum PSA values <4 ng/mL at time of biopsy and have been cancer positive. This suggests that there is no sufficient PSA cutoff which allows excluding PCa. Thompson et al. and our study group demonstrated this fact in former studies [19, 20]. 

**Figure 4:**
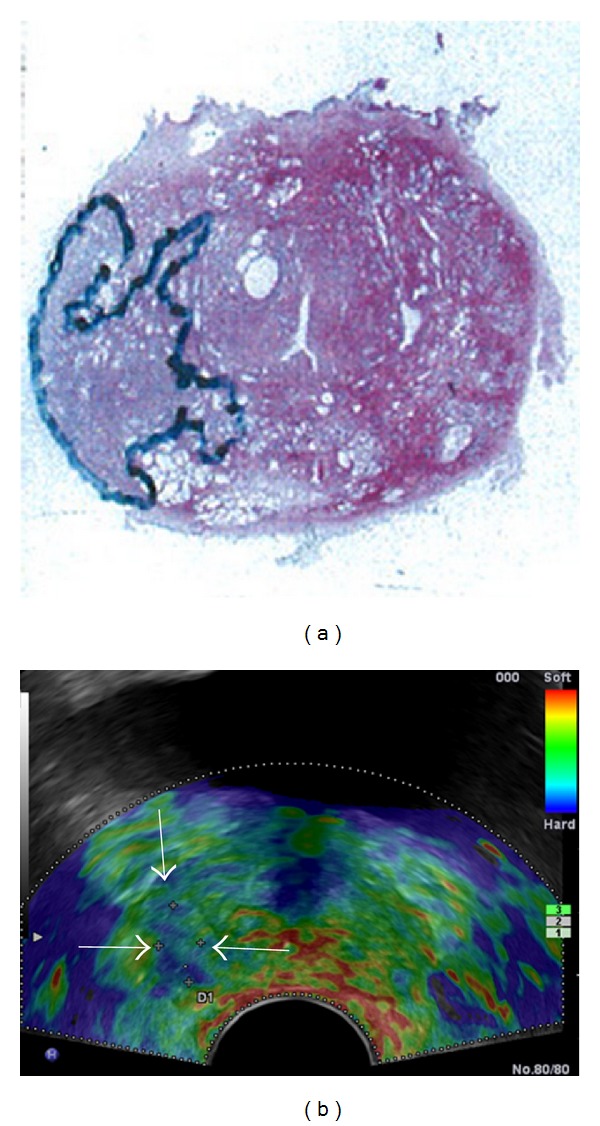
Outlined large cancer lesion PZ midgland right on whole-mount step section shown on (a) and corresponding hard area (white arrows) on elastogram (b) in axial planes.

Therefore, imaging modalities to visualize PCa should raise the confidence for PCa detection independently of serum PSA values and should demonstrate significant cancer disease with high sensitivities. Detecting insignificant disease may lead to overdiagnosis and overtherapy [3]. Some authors define cancer lesions <0.5 cm^3^ as insignificant, whereas other prefer a treshold of <0.2 cm^3^ [10, 11]. In our series, RTE was capable to demonstrate 83.3% of all cancer lesions with a tumor volume ≥0.2 cm^3^ and 91.2% with a tumor volume of ≥0.5 cm^3^ (Figure 4).

**Figure 5:**
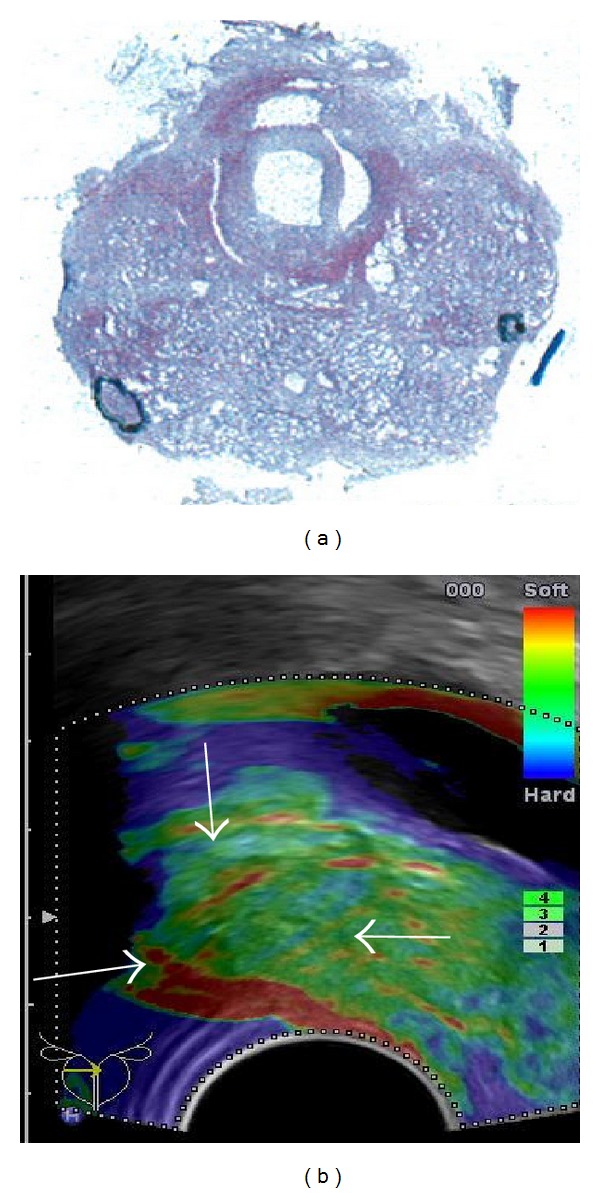
Outlined small cancer lesion PZ base right on whole-mount step section shown on (a) and corresponding elastogram on (b) in axial plane with arrow marked soft base.

Regarding the largest diameter the detection rate in the group 0–5 mm was weak with 9.7%, also not satisfying in the group 6–10 mm with 27% (Figure 5). However, as stated above: should we really be able to detect those small cancer lesions?

Roethke et al. investigated tumor size dependent detection rates of well-established T2 weighted magnetic resonance imaging (T2w-MRI) and found sensitivities of 45% and 89% for lesions with a maximum size of 10–20 mm and >20 mm, which is slightly lower than our results (70.6% and 100%; resp.) [21]. Furthermore, they concluded that T2w-MRI cannot exclude PCa with lesions smaller 10 mm and 0.4 cm^3^ and that including foci smaller 10 mm or less than 0.5 cm^3^ decreased sensitivity clearly. Similar to our results, the presented data suggest that generally imaging of PCa is limited due to tumor size. Nevertheless, they considered their detection rate for lesions more than 20 mm (1.6 cm^3^) as high [21].

In contrast, Walz et al. concluded that RTE alone did not allow the identification of the PCa index lesion with satisfactory reliability, which should be necessary for focal therapy. They compared RTE findings with whole-mount step sections to evaluate the diagnostic accuracy for identifying the PCa index lesion, which is considered to be responsible for possible metastatic progression and cancer-specific death and observed a sensitivity of only 58.8% [4]. 

Sumura et al. reported sensitivities for RTE of 72.7% for tumors with volume <0.1 cm^3^, 77.8% for tumors with volume 0.1–0.3 cm^3^, 71.4% for tumors with volume >0.3 cm^3^, and 100% for tumors with volume >0.5 cm^3^ [22]. Similar to our study, the detection rates for both anterior and posterior tumors were nearly equal. Furthermore, our data indicate that PV and PSA serum values have no significant influence for detection rates in significant disease (Table 1). 

Nevertheless, we missed 8 of 48 cancer lesions with a tumor volume >0.2 cm^3^ on RTE, which means nearly 20% of significant disease. Our pathologist reevaluated the whole-mount step sections of these 8 cases and all missed cancer lesions had a predominant Gleason pattern 3 and no cancer lesion with predominant Gleason pattern of 4 or 5 was missed. 

**Figure 6:**
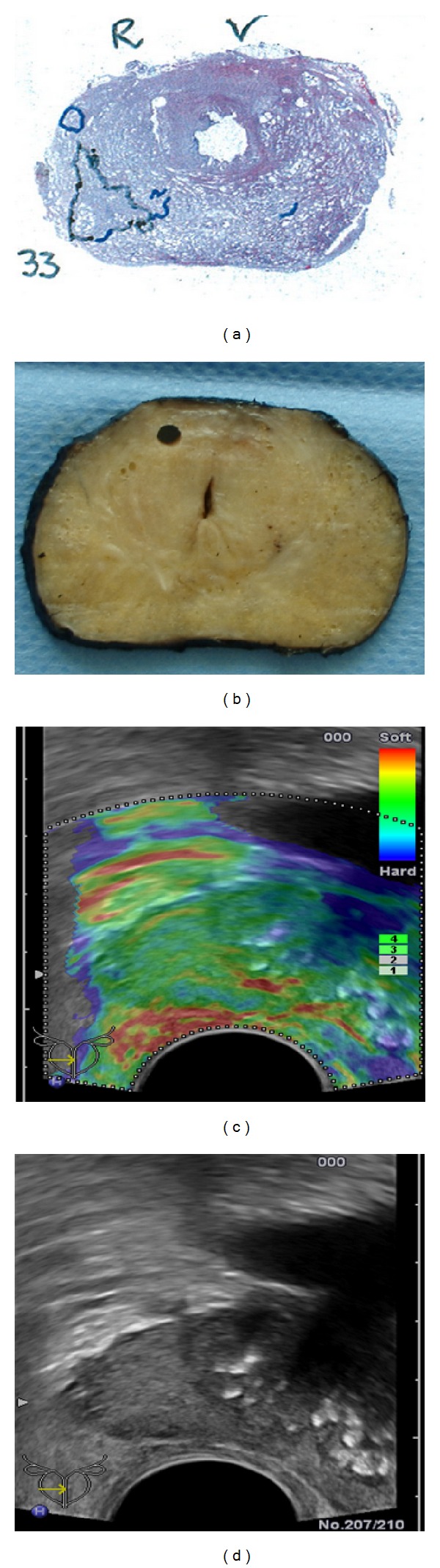
Outlined sparse cancer lesion PZ base right on whole-mount step section (a), no suspicious changes on macroscopic specimen (b), elastogram (c), and grey-scale ultrasound (d).

In total, 6 of the 8 cancer lesions showed sparse architecture on histology, which means they were intermixed with normal glands and also with glands showing dilated lumina and so are more fluid and therefore soft which is a limitation for RTE (Figure 6). The 2 other cases were dense tumors and had volumes of 0.22 cm^3^ and 0.41 cm^3^. This fact was also described by Langer et al. who investigated the outcome of diffusion weighted MRI (DWI) and T2w-MRI in dependence of histological tumor composition [23]. Similar to RTE, DWI assesses tissue information due to cell density: the denser the cancer, the higher the diffusion restriction. All of their “invisible” tumors also had predominant Gleason pattern 3 and showed sparse architecture on histology, so that they did not significantly differ from healthy prostate tissue. The authors concluded that this may limit T2w-MRI and DWI in the detection and the assessment of tumor volume of some cancers.

Some cancer lesions may be negative on RTE, but positive on CE-TRUS [20]. Maybe this multiparametric way—adding tissue informations about contrast media dynamics to grey-scale ultrasound and RTE—could have detected some of our false negative findings. Brock et al. have shown the usefulness of this approach, whereas Nygård et al. demonstrated the benefit of adding new biomarkers, like PCA-3, to RTE findings for the detection of significant disease [24, 25].

Our study has several limitations. (1) We do not have data about intra- and interobserver variability. (2) We have used only one US system for RTE. The reproducibility of our results with other US systems needs to be evaluated in further studies. (3) We focused this study on correlating tumor sizes; we did not correlate right negative results between RTE and histopathologic specimens. (4) We investigated the PZ only due to the above mentioned reasons. (5) We knew that every patient had PCa, which is a bias. (6) The planes of whole-mount step sections had an orientation of 90° to the urethra, while an endfire transducer provides images in different angles. Therefore it could be difficult to be sure, whether the identical geographic areas were compared. An investigation with 3D/4D ultrasound would be desirable. 

## 5. Conclusion

RTE is capable of detecting significant PCa with high sensitivity, but can have problems when visualizing tumors with sparse architecture. Therefore, adding information about contrast media dynamics in a multiparametric way may decrease the number of false negative cases. In the detection of smaller cancer lesions, RTE is of limited value.

## Figures and Tables

**Figure 2:**
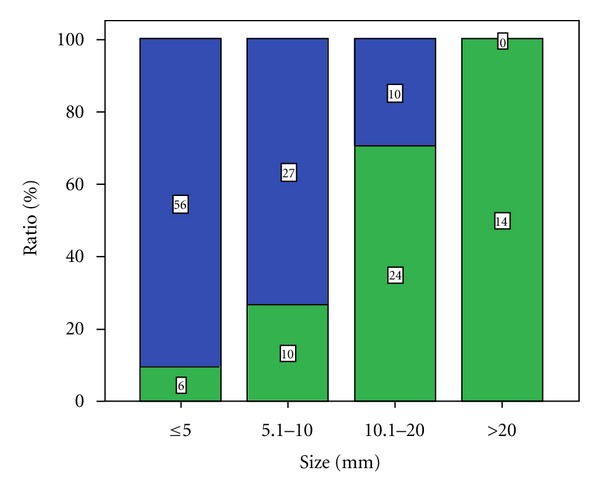
Detection rate based on tumor size.

**Figure 3:**
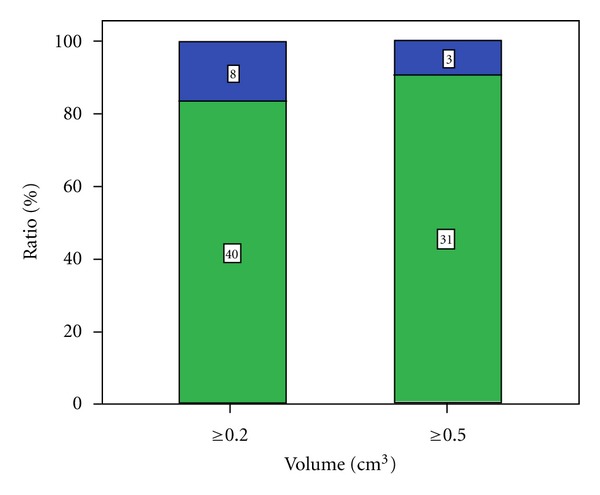
Detection rate based on tumor volume.

**Table 1:** Detection rate of all lesions ≥0.2 cm³ in dependence of localization, Gleason scores, prostate volumes and PSA serum values; *n* = 48.

	Detection	*P*
Localization		
Anterior	66.7% (6/9)	0.419
Posterior	80% (20/25)	
Both	100% (14/14)	

Gleason score		
G5, G6, G7 (3+4)	75% (24/32)	0.028
G7(4+3), G8, G9, G10	100% (16/16)	

PV (mL)		
<40	86.8% (33/38)	0.204
≥40	70% (7/10)	

PSA (ng/mL)		
<4	84.6% (11/13)	0.885
≥4	82.9% (29/35)	
